# Fatigue, Health and Mortality from age 70-100

**DOI:** 10.1093/geroni/igaf122.163

**Published:** 2025-12-31

**Authors:** Irit Stessman-Lande, Aliza Rozenberg, Jochanan Stessman, Jeremy Jacobs

**Affiliations:** Hadassah University Medical Centers; The Department of Geriatric Rehabilitation, Jerusalem, Yerushalayim, Israel; Hebrew University of Jerusalem, Hadassah Medical Center, Jerusalem, Yerushalayim, Israel; Hebrew University of Jerusalem, Hadassah Medical Center Faculty of Medicine, Jerusalem, Yerushalayim, Israel; Hadassah Hebrew University Hospital, Jerusalem, Yerushalayim, Israel

## Abstract

Fatigue is common with aging, yet poorly described. We examined the prevalence, health and functional status, and mortality associated with fatigue between ages 70-100. The Jerusalem Longitudinal Study (1990-2021) prospectively followed a community-dwelling cohort born 1920-21, at ages 70, 78, 85, 90, 95 and 100 (n = 604, 1024, 1222, 729, 508, 205 respectively). Comprehensive assessment included fatigue (“Do you feel generally tired?”) and mortality data. Logistic regression models (adjusted for gender, education, self-rated health [SRH], Charlson comorbidity index [CCI], ADL, depression, chronic musculoskeletal pain), Kaplan-Meier survival curves, and Cox proportional hazards models (adjusted for gender, education, CCI) were performed. At ages 70, 78, 85, 90, 95, 100 the prevalence of fatigue was 29.4%, 52.3%, 67.9%, 77.2%, 64.9%, and 65.6%. Between age 70-100, fatigue was significantly associated with numerous cross-sectional health and functional measures, and subsequent deterioration for loneliness, SRH, ADL dependence, and physical activity. In contrast, no consistent pattern of association with current or subsequent common medical morbidities was observed. Significantly decreased survival rates were observed among fatigued vs. non-fatigued subjects at ages 70-78, 78-85, 85-90, 90-95: 69.6% vs 80.8% (p = 0.0078), 64.9% vs 73.3% (p = 0.0016), 60.6% vs 76.4% (p < 0.0001), 51.3% vs 65.0% (p = 0.0068) respectively, yet not 95-100: 21.7% vs 18.8% (p = 0.99). After adjusting for known mortality risk factors, fatigue at age 70, 85 and 90 was associated with increased mortality. Fatigue is increasingly common with advancing age, and demonstrates significant negative impact upon health and survival. The poor association with medical morbidities raises questions concerning possible pathways of action.

